# Proton–deuterium exchange of acetone catalyzed in imidazolium-based ionic liquid–D_2_O mixtures

**DOI:** 10.1039/d0ra04206d

**Published:** 2020-09-02

**Authors:** Astghik A. Shahkhatuni, Aleksan G. Shahkhatuni, Suren S. Mamyan, Valentine P. Ananikov, Arpine S. Harutyunyan

**Affiliations:** Molecule Structure Research Center, STCOPC NAS RA Azatutian Ave. 26 Yerevan 0014 Armenia astriksh@gmail.com; Zelinsky Institute of Organic Chemistry, Russian Academy of Sciences Leninsky Prospect 47 Moscow 119991 Russian Federation

## Abstract

The reaction of the proton–deuterium exchange of acetone in imidazolium-based ionic liquid (IL)–deuterium oxide mixtures was studied in detail *via* NMR spectroscopy. Certain ILs exhibit considerable catalytic properties and contribute to the course of reaction up to the complete deuteration. The efficiency of deuterium exchange crucially depends on the features of ILs; the type of anion and chain length of cation. The linear secondary isotope effects on the NMR chemical shifts of the ^13^C atoms in acetone were observed depending on the deuteration level of the molecule.

## Abstract 2

The reaction of the proton–deuterium exchange of acetone in imidazolium-based ionic liquid (IL)–deuterium oxide mixtures was studied in detail *via* NMR spectroscopy.

## Graphical abstract

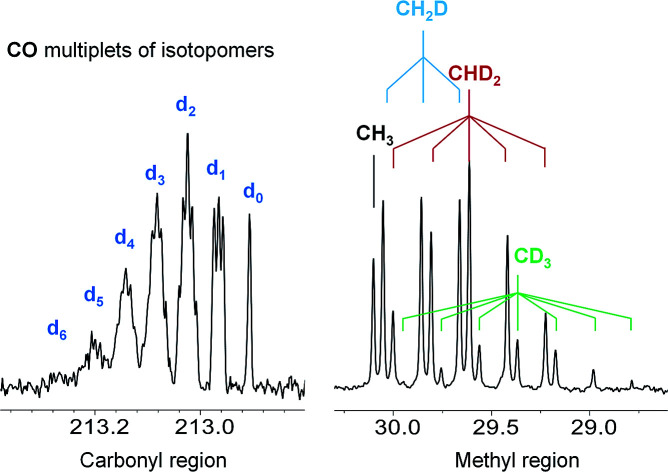

## Introduction

Efficient deuterium labelling of organic compounds became a topic of outstanding importance in recent years because of the improved pharmacokinetic properties demonstrated by the D-labelled drugs.^1–5^ On the one hand, the properties of C–H and C–D bonds are very similar, which allows for keeping the desired biological activity of a drug molecule upon C–H/C–D replacement. On the other hand, the C–D bond is stronger than the C–H bond,^6^ which is the key characteristics to improve the drug efficiency by protecting oxidatively labile C–H bonds or *via* other mechanisms.^1–5^ The approval of the first deuterated drug on the market opened a new demand for the development of new synthetic methodologies and their mechanistic understanding.^7^

From the synthetic point of view, the replacement of C–H/C–D reactions can be carried out using transition metal catalysis. However, the application of the metal catalyst leads to the unavoidable contamination of drug molecules with metal traces and requires rigorous purification before use. Therefore, metal-free organocatalytic approaches are of considerable importance to develop new synthetic applications.^8–14^ N-Heterocyclic-carbene-based organocatalytic reactions and ionic liquids have been used actively in this area.^15–18^

The deuterium exchange reaction of H atom in the second position in the imidazolium ring of imidazolium-based ionic liquids (ILs) mixtures with deuterium oxide is a well-known process with promising opportunities to transfer the D-label to organic functional groups. It was shown that depending on the IL the rate and overall level of deuteration can change.^19–25^ Our studies also showed that under the same conditions, the degree of deuteration in different ILs was different ranging from complete deuteration to its absence. Of particular interest are IL/water mixtures, where micro-heterogeneous structures induce new chemical properties^26^ and effect the biological activity.^27,28^ The H/D exchange can occur in molecules of compounds dissolved in IL–water systems as well; however, this phenomenon is less studied and its practical potential is not fully utilized. It was shown that mixtures of some ILs with CDCl_3_ are active catalysts for the H/D exchange reaction of ketones and alkyne substrates.^29,30^ Recently, a dynamic nature of imidazolium systems was revealed and ambident reactivity with carbonyl compounds was shown in organocatalytic processes.^31^ Reactions involving ketones are of particular interest with challenging questions from the mechanistic point of view.^31^ Acetone, despite being a small molecule, was established as a reliable model for studying the deuteration reactions of ketones and gain mechanistic understanding.

Deuterium exchange in the keto–enol tautomeric equilibrium reactions of acetone in heavy water catalyzed by bases is being studied for a long time.^32^ The process can occur in deuterated solvents, having exchangeable groups, such as CDCl_3_, methanol–OD, ethanol–OD or heavy water D_2_O. Moreover, this reaction was used for the measurements of the ^2^H-labelling of water in biological fluids, which are required for determining the rates of the biochemical flux and for estimating the body composition.^33,34^ However, for such cases the base-catalyzed exchange of hydrogen (deuterium) between water and acetone is used, and the ^2^H-labeling of acetone is then determined *via* gas chromatography-(quadrupole) mass spectrometry. Without the catalyst, in pure CDCl_3_ or D_2_O solution of acetone, the H/D exchange reaction does not take place.

However, it is known that in CDCl_3_ in the presence of 1,2,3-trialkylimidazolium-based ionic liquids associated with basic anions such as hydrogen carbonate, prolinate, and imidazolate the H/D exchange reaction of various substrates occurs, without the addition of any extra bases or metal.^30^

Here, we decided to study the effect of other ILs on the H/D exchange processes of acetone, using D_2_O as the D source. We report the deuteration of methyl groups in acetone, one of the simplest ketonic compounds, dissolved in IL-D_2_O mixtures, with the aim of understanding if these mixtures could serve as catalysts and/or hinder the deuterium exchange in solute molecules. The ketone functional group is ubiquitously present in drugs and biologically active molecules. Gaining a mechanistic insight into the C–H/C–D replacement is an important area for the development of IL-mediated metal-free D-labelling reactions.

## Experimental

**Fig. 1:**
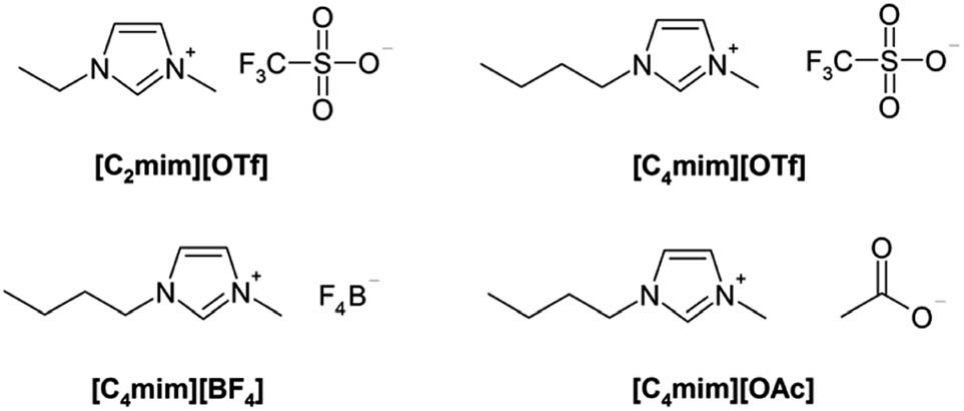
Structures of the used ILs.

ILs used in the study (Table 1, and Fig. 1) were obtained from ABCR, Germany, and Acros, Belgium. D_2_O (99.9 atom% D) was purchased from Cambridge Isotope Laboratories. Commercially available acetone was purified before use.

**Table 1:** Summary of the ILs used in this study

Abbreviation	Name
[C_4_mim][BF_4_]	1-Butyl-3-methylimidazolium tetrafluoroborate
[C_4_mim][OAc]	1-Butyl-3-methylimidazolium acetate
[C_4_mim][OTf]	1-Butyl-3-methylimidazolium trifluoromethanesulfonate
[C_2_mim][OTf]	1-Ethyl-3-methylimidazolium trifluoromethanesulfonate

Composition of all samples in D_2_O is 4.0% v/v of acetone, with 1/1 v/v D_2_O/IL. The composition of the sample in H_2_O is 4.0% v/v of acetone-d_6_, with 0.9/1 v/v H_2_O/[C_4_mim][OAc], so that the samples with H_2_O and D_2_O will have the same molar ratio of water/[C_4_mim][OAc].

The ^1^H and ^13^C inverse gated NMR spectra of the samples presented here were acquired at 303 K on a 400 MHz Bruker AVANCE NEO spectrometer equipped with a temperature controlled Smart probe. DEPT (Distortionless Enhancement by Polarization Transfer) and APT (Attached Proton Test) NMR techniques were used to clearly represent the level of the deuteration of methyl groups and were acquired also on a Varian Mercury 300 VX. MestreNova was used to process spectra.

## Results and discussion

Acetone has seven possible H/D mass isotopomers (with masses ranging from 58.080 for unlabeled two CH_3_ groups to 64.116 for fully labelled CD_3_ groups), and ten isotopomers, considering the labelling positions. The distribution among them, rate and level of deuteration depend on the temperature, acetone concentration, IL–D_2_O ratio, and type of IL.

**Fig. 2:**
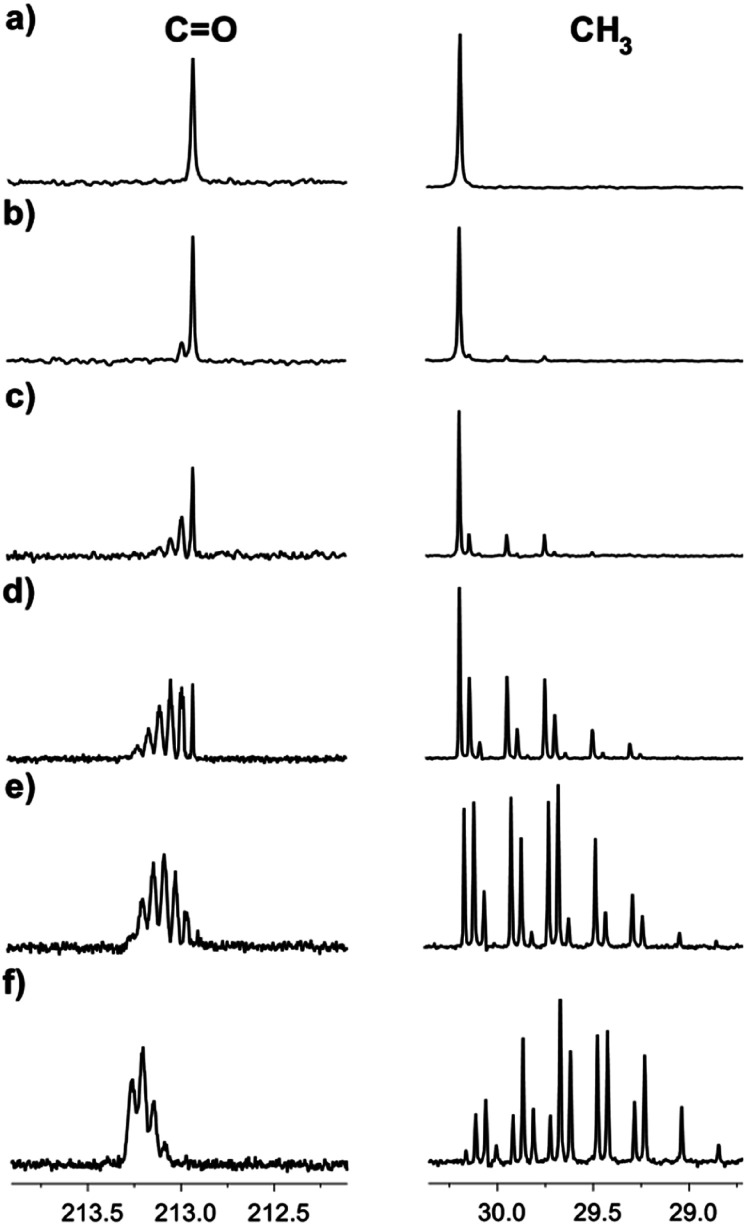
^13^C NMR spectra of acetone in the binary mixtures of D_2_O in the IL [C_4_mim][OAc] after a certain reaction time: (a) one hour; (b) 1 day, % D = 1.5; (c) 1 week, % D = 7.5; (d) 3 weeks, % D = 20; (e) 5 weeks, % D = 34; (f) 4 months, % D = 81.

^13^C NMR spectroscopy provides an excellent possibility to study the H/D exchange due to the existing large isotope effects on the chemical shifts of carbons in acetone, leading to the separation of signals, corresponding to various isotopomers. The deuteration can be easily detected due to the splitting patterns arising from the deuterons attached to the carbon atoms both in the ^1^H and in ^13^C{^1^H} NMR spectra (deuterium is an isotope with spin = 1). Moreover, the study can be done by monitoring the signals of both carbonyl and methyl groups. The sequential deuteration of CH_3_ groups of acetone in numerous IL–D_2_O mixtures is a slow process at room temperature and can be easily monitored. Deuterium enrichment (in %) will be estimated by the degree of deuteration % D.^10^ As an example, in Fig. 2, the H/D exchange of acetone in [C_4_mim][OAc]-D_2_O at various time intervals of the reaction is presented (the reaction begins directly in the NMR tube after sample preparation). After one hour of reaction, there is no visible deuteration and the spectrum of acetone is seen, consisting of two singlets, corresponding to carbonyl and methyl carbons (Fig. 2a). The deuteration was evident in the NMR spectra in this sample even after one day (Fig. 2b). As time passes, the isotopomer signals begin to appear as a result of sequential deuteron-exchange (Fig. 2b–f).

**Fig. 3:**
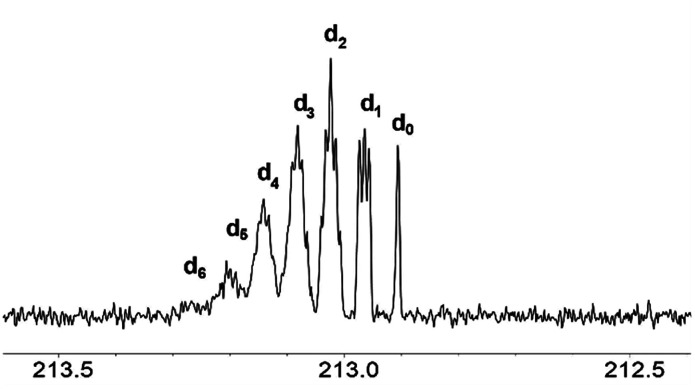
^13^C NMR spectra of the CO region of acetone in the binary mixtures of D_2_O with IL [C_4_mim][OAc] after 3 weeks of reaction (% D = 28).

The signals of the carbonyl group of the mixture of acetone isotopomers appear in the region of 212.5–213.5 ppm. Theoretically, there could be seven multiplets, corresponding to seven mass isotopomers with a different deuterium content in the molecule of acetone. On the ^13^C{^1^H} 400 MHz NMR spectra the signals of the isotopomers moved downfield from the singlet signal corresponding to the unlabelled acetone (Fig. 3). They are ordered by the number of labelled deuterium atoms in each isotopomer and separated from each other by Δ*δ* = 0.0588 ppm. The first multiplet is a 1:1:1 triplet and corresponds to acetone-d_1_, the second multiplet is a 1:2:3:2:1 quintet and corresponds to acetone-d_2_, followed by 1:3:6:7:6:3:1 septet (acetone-d_3_), then 1:4:10:16:19:16:10:4:1 nonet (acetone-d_4_), then 1:5:15:20:45:51:45:20:15:5:1 undectet (acetone-d_5_), and finally 1:6:21:40:80:116:141:116:80:40:21:6:1 tredectet (acetone-d_6_).

Signals of most of the isotopomers with their fine structures are shown in Fig. 3. Each of the d_2_, d_3_ and d_4_ signals corresponds to two isotopomers with differently labelled methyl groups, but since they have very close chemical shifts and small spin–spin coupling constants over two bonds ^2^*J*_CD_ = 0.8 Hz, the spin system can be considered as AX_*n*_, where *n* is a number of labeled deuteriums. Due to the above-mentioned, not all lines in all of these multiplets are well resolved. Obviously, the further the H/D reaction goes, the more intensive are the d_5_ and d_6_ signals, and the d_0_–d_2_ disappear (Fig. 2f).

The ^13^C{^1^H} spectral patterns of the methyl groups in the acetone isotopomers appear in the region of 29.0–31.0 ppm and also allow to distinguish the isotopomers by the number of deuterons attached to the carbon (CD = 1:1:1 triplet, CD_2_ = 1:2:3:2:1 quintet, CD_3_ = 1:3:6:7:6:3:1 septet). As the deuteration progresses, the triplet corresponding to the CH_2_D group appears (Fig. 2b) in addition to the singlet line corresponding to the non-deuterated CH_3_ group (Fig. 2a). After a week of the H/D exchange reaction, the quintet with low intensity, corresponding to the CHD_2_ group becomes also visible (Fig. 2c). After three weeks of the reaction, the intensities of the CH_2_D triplet and CHD_2_ quintet has grown significantly, while the intensity of the CH_3_ singlet has decreased. After five weeks of reaction, the signals of the CD_3_ septet have started also to appear (Fig. 2e–f).

**Fig. 4:**
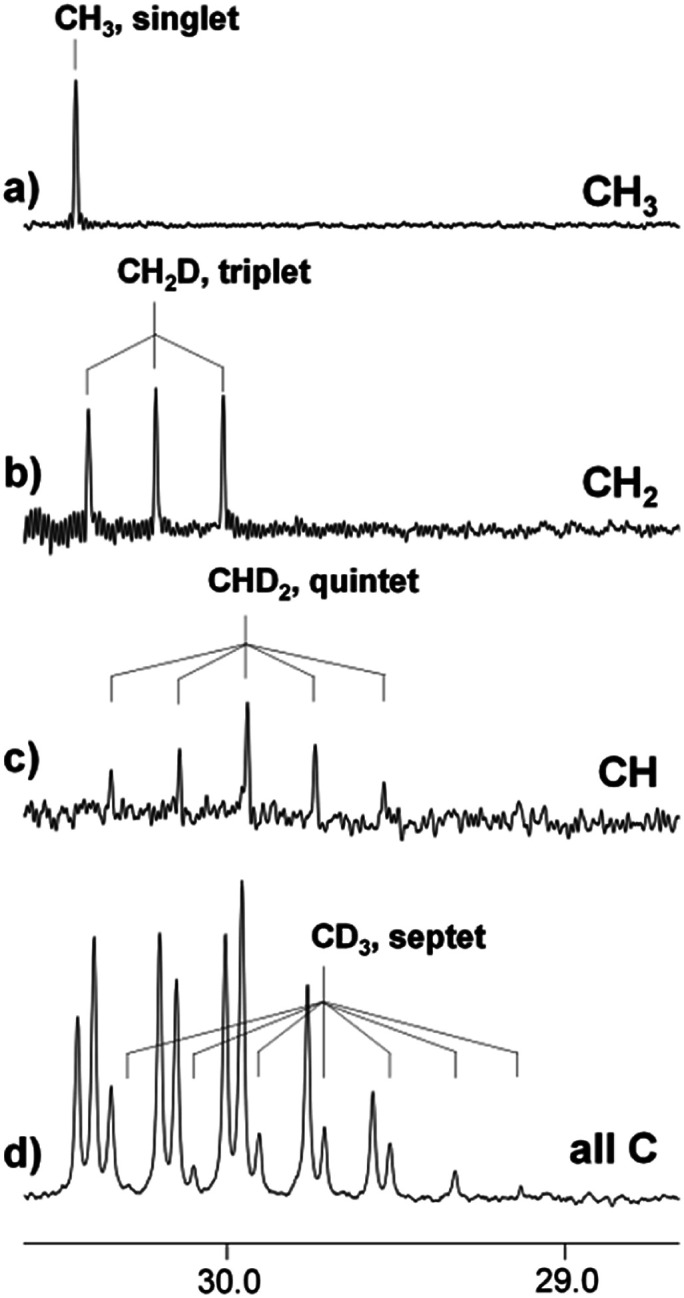
^13^C NMR DEPT (a–c) spectrum and ^13^C{H} (d) spectra of acetone in the [C_4_mim][BF_4_]–D_2_O binary mixture after 5 months of reaction (% D = 40).

Thus, the proportions of isotopomers with different ratios of deuterated groups coexist in one sample, which can be clearly visualized by the DEPT experiment. DEPT is an experimental NMR technique, which allows to determine the number of hydrogens bound to each carbon. The DEPT spectrum consists of three separate spectra, corresponding to the carbon atoms of CH_3_, CH_2_, and CH groups, or, in our case, to the CH_3_, CH_2_D, and CHD_2_ groups (Fig. 4a–c). Signals from the CD_3_ group with no attached protons are absent in the DEPT spectra. The spectrum in Fig. 4d is the proton decoupled ^13^C spectrum, in which signals of all carbons are visible including the septet arising from the CD_3_ group.

**Fig. 5:**
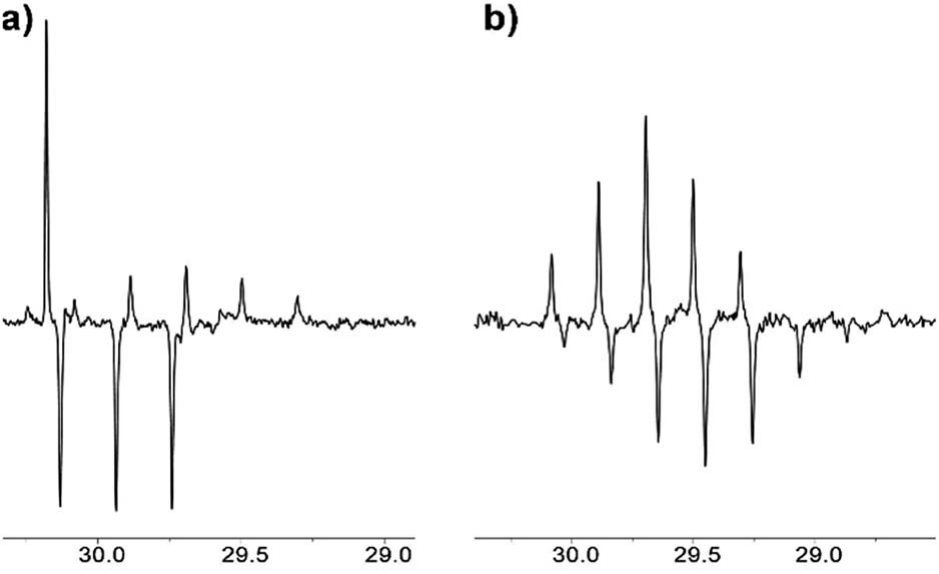
^13^C NMR APT spectrum of acetone in [C_2_mim][OTf]–D_2_O binary mixture after (a) 1.5 weeks, % D = 23; (b) 5 months of reaction, % D = 90.

Analogously, the composition of the reaction mixture can be monitored by the APT NMR experiment (Fig. 5), which was acquired in this work using Bruker Avance NEO spectrometer.

In the APT spectrum, signals from the carbons with an odd number of protons are in antiphase with those containing even number of protons. After 1.5 weeks in [C_2_mim][OTf]-D_2_O, the singlet and quintet corresponding to the CH_3_ and CHD_2_ groups and the triplet from the CH_2_D group can be seen as positive and negative signals, respectively (Fig. 5a). Finally, after 5 months of reaction, singlet and triplets signals corresponding to the CH_3_ and CH_2_D groups fully disappear from the NMR spectra, and only signals corresponding to the CHD_2_ and CD_3_ groups are visible in the spectrum, with a quintet appearing upfield (*i.e.* positive), and a septet appearing downfield (Fig. 5b).

Thus, in the IL–D_2_O mixture it is possible to find many (up to all possible) isotopomers of acetone with a certain distribution by the level of deuterium labelling after a certain period of reaction time.

**Fig. 6:**
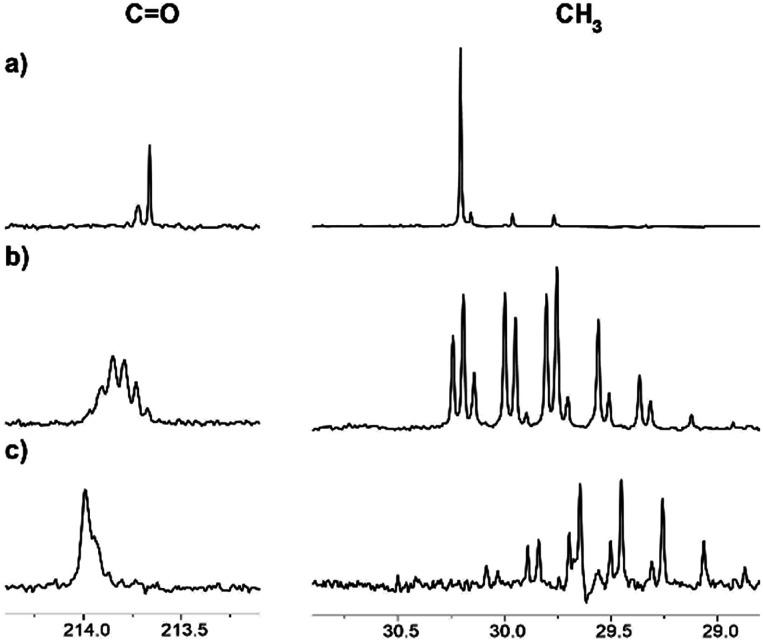
^13^C NMR spectra of acetone in the binary mixtures of D_2_O with ILs after 5 months of reaction: (a) [C_4_mim][OTf], % D = 4; (b) [C_4_mim][BF_4_], % D = 40; (c) [C_2_mim][OTf], % D = 95.

Interestingly, we have observed that the rate of the deuteration process is essentially different for various ILs (Fig. 6). The reaction in the [C_4_mim][OAc] and [C_2_mim][OTF] systems occurred relatively faster, with almost complete deuteration of acetone in the latter observed in 5 months (Fig. 6c). The slowest process takes place in the mixture of [C_4_mim][OTf], where even after 5 months the deuteration did not go further than the CH_2_D group (Fig. 6a). Such different deuteration efficiencies in the [C_2_mim][OTF] and [C_4_mim][OTF] samples imply that the addition of only one CH_2_ group to the alkyl chain of the imidazolium ring changes drastically the effect of IL on the deuterium exchange processes. On the other hand, the exchange in the [C_4_mim][BF_4_] sample (Fig. 6b) is greater than that in [C_4_mim][OTf], and less than that in [C_4_mim][OAc] in the same time interval, which means that the nature of the anion also has a significant effect and should be taken into account.

**Fig. 7:**
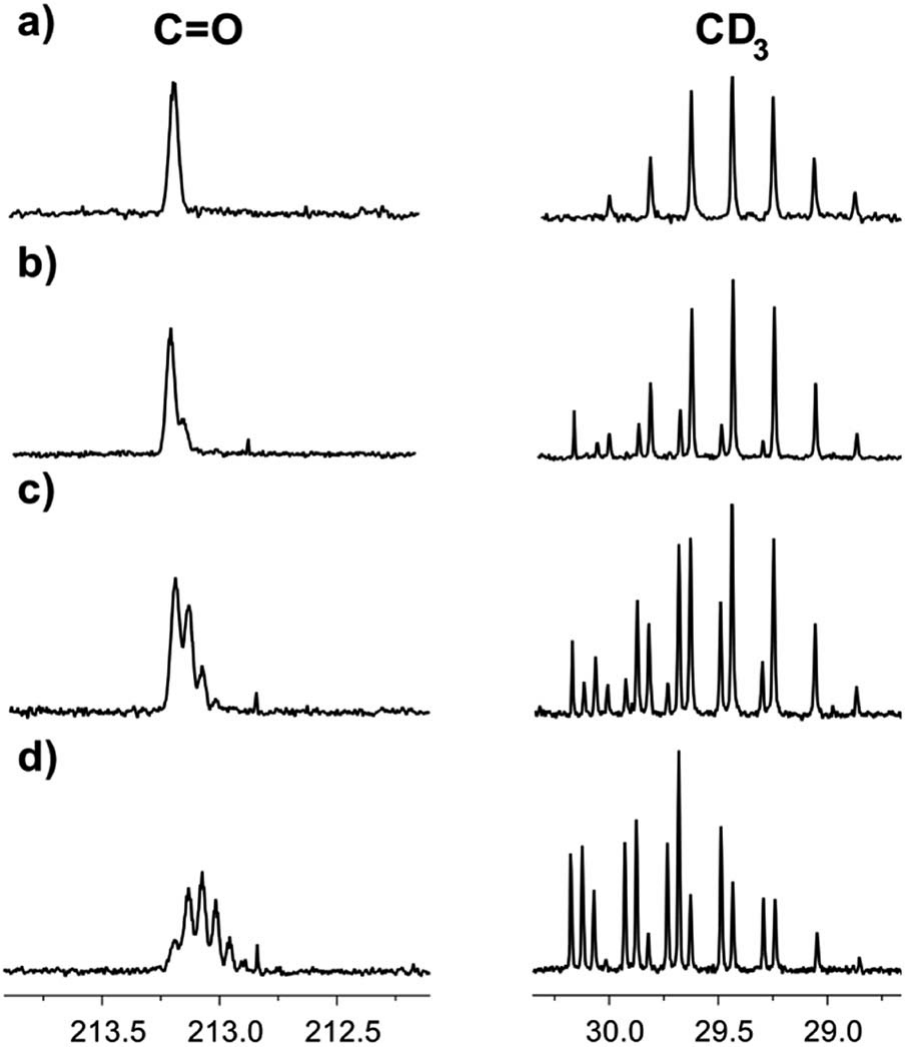
^13^C NMR spectra of acetone-d_6_ in binary mixtures of H_2_O with IL [C_4_mim][OAc] (a) the same day, % D = 100; (b) 2 days later, % D = 96; (c) 5 days later, % D = 88; (d) 16 days later, % D = 67.

Correspondingly, the opposite process of the D/H exchange is also possible, if a mixture of acetone-d_6_ and H_2_O is prepared.^13^ We have studied acetone-d_6_ in H_2_O and in the [C_4_mim][OAc]–H_2_O mixture. In pure H_2_O, there was no hydrogen replacement by deuterium after at least five weeks, as opposed to the [C_4_mim][OAc]–H_2_O mixture, where the D/H exchange becomes visible the next day and the rate of hydrogen transfer is quite high (Fig. 7). As expected, the appearance of consecutive multiplets is in the reverse order compared to the H/D exchange pattern.

**Fig. 8:**
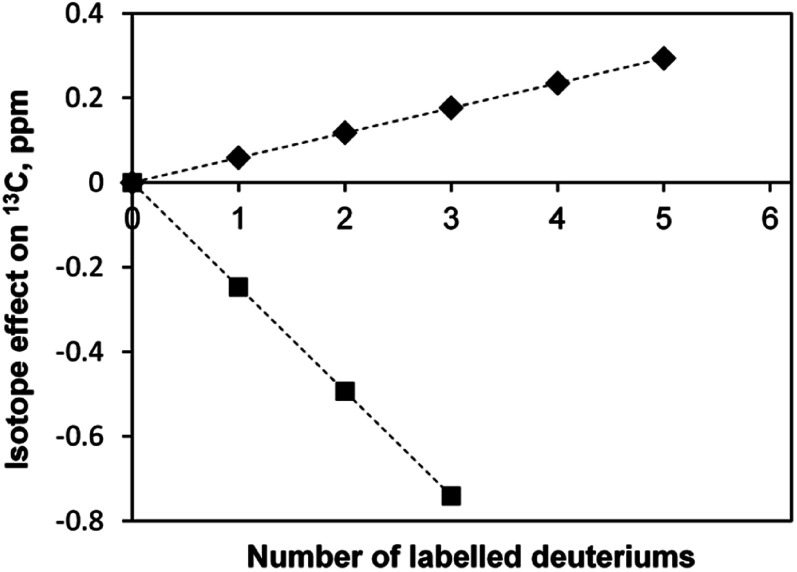
Deuterium isotope effects on the chemical shifts of ^13^C carbons of acetone in [C_4_mim][BF_4_]; ◆ – carbonyl group, ■ – methyl group.

It is known that there are deuterium isotope effects on the NMR chemical shifts and spin–spin coupling constants (see, for instance, the case of dichloromethane^35^). We have also observed such isotope effects. For instance, in the sample [C_4_mim][BF_4_]-D_2_O the secondary linear upfield deuterium isotope effects on the chemical shifts (in ppm) of the ^13^C-methyl atoms of acetone depending on the deuteration level *n*, equals to −0.2469 × *n*, and the downfield linear isotope effect on the ^13^C-carbonyl atom, equals to 0.0587 × *n* (Fig. 8). A primary isotopic effect of −0.068 × *n* (in Hz) on the ^1^*J*_CD_ spin–spin coupling constants of methyl carbon is also observed.

## Conclusions

Here, we described a unique phenomenon of the catalytic H/D-exchange reaction in acetone mediated by imidazolium-based ILs. We have studied the deuteration of the methyl groups of acetone in D_2_O aqueous mixtures of several imidazolium based ILs *via* NMR spectroscopy. Depending on the type of anion and/or alkyl chain length of the cation, the effectiveness of the H/D exchange differs significantly. The shorter the chain the more effective is the exchange. An almost complete deuteration was observed in the presence of [C_4_mim][OAc] and [C_2_mim][OTf].

The linear deuterium isotope effects on the NMR chemical shifts of ^13^C atoms of acetone were observed depending on the deuteration level of the molecule (upfield for methyl carbon, and downfield for carbonyl).

Obviously, the observed phenomenon opens several new directions for further studies. Here, we focused our investigation on the room temperature reaction to reveal the process in complete details and to detect all the possible isotopomers with their interconversions. Increasing the temperature to speed up the process and enlarging the scope to involve different ketones will be a subject for our future studies.

## Conflicts of interest

There are no conflicts to declare.

## Supplementary Material
